# Epithelioid Angiosarcoma Causing Spinal Cord Compression

**DOI:** 10.7759/cureus.14325

**Published:** 2021-04-06

**Authors:** Luis F Lemus, Mario H Minervini

**Affiliations:** 1 Department of Surgery, Universidad Dr. José Matías Delgado, San Salvador, SLV; 2 Radiosurgery, International Cancer Center, Diagnostic Hospital, San Salvador, SLV

**Keywords:** epithelioid angiosarcoma, extradural tumor, cervical spine, endothelial tumor

## Abstract

Epithelioid angiosarcoma is a rare and very aggressive malignant tumor with high rates of metastasis and recurrence that can present in any part of the body, with the head and neck being the most common regions. Wide-margin surgical resection is the treatment of choice following radiotherapy due to the high rate of recurrence. We present a case of an elderly patient who developed angiosarcoma causing spinal cord compression at the level of C7 vertebrae. We discuss the diagnosis, treatment, histopathology, and outcome.

## Introduction

Epithelioid angiosarcoma is a rare and highly aggressive endothelial cell malignant tumor with a high mortality rate [1]. Although they often arise in sun-exposed skin (60% of the cases) and deep soft tissues such as the head and neck, they have been reported in other sites such as the breast, thyroid, kidney, bone, adrenal gland, vagina, testis, liver, and spleen [1-5]. Angiosarcoma is a tumor with a high rate of recurrence and metastasis. The reported percentage of advanced disease varies from 16% to 44%, and the overall survival varies from six to sixteen months [6].

As angiosarcoma is rare, most of the information known about the disease comes from case reports or small cohort studies with small sample sizes. Here, we present a case report that highlights an unusual presentation of angiosarcoma [7]. Physical examination, clinical course, radiological features, pathological findings, and treatment are discussed.

## Case presentation

**Figure 1:**
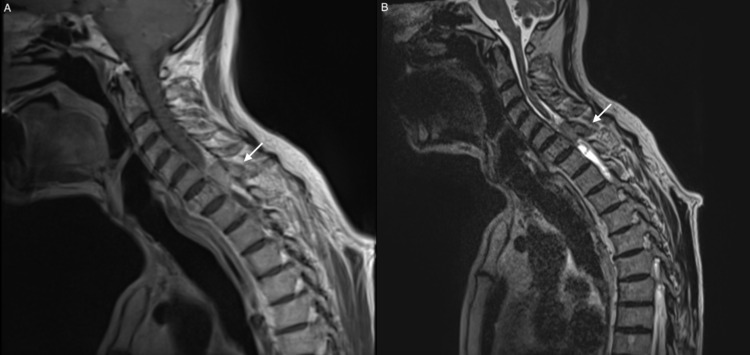
Sagittal MRI of the spine. (A) T1-weighted sagittal MRI shows injury of the vertebral body of C7 and presence of a non-enhancing extramedullary tumor measuring 4.19 cm. (B) T2-weighted sagittal MRI shows a hypointense extramedullary tumor MRI, magnetic resonance imaging

**Figure 2:**
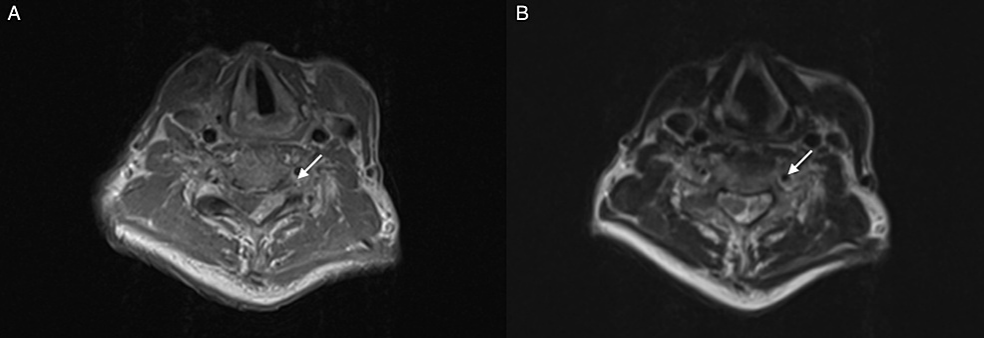
Axial MRI of the spine. (A) T1-weighted axial MRI shows an extramedullary tumor on the left side of the spinal cord causing compression and displacement to the right. (B) T2-weighted axial MRI shows an extramedullary tumor with enhancement of paravertebral soft tissues. MRI, magnetic resonance imaging

A 69-year-old male presented to the clinic with complaints of weakness in the left lower limb for the last four months followed by gradual numbness. In the past two months, the symptoms progressed to both lower and upper limbs and the patient had difficulty walking. On neurological examination, the patient had muscle weakness in both upper and lower limbs (motor strength 3/5), hyperreflexia (+3) in both patellar and Achilles reflexes, increased muscular tone in lower limbs, bilateral Babinski sign, alteration in tactile sensitivity, and perception of pain and temperature from the level of the nipple to the feet. Magnetic resonance imaging (MRI) showed a mass at the level of the vertebral body of C7 with the presence of an extramedullary tumor measuring 4.19 cm with infiltration of the corresponding articular facets and tumor exits through neural hole C7-T1 (Figure 1). Axial images demonstrated the presence of an extramedullary tumor on the left side of the spinal canal causing spinal compression and displacement of the cord to the right (Figure 2).

**Figure 3:**
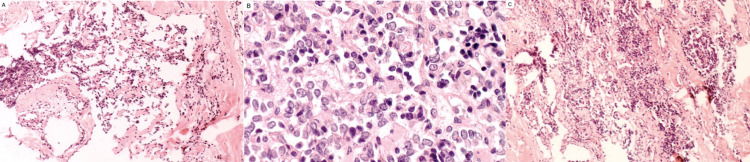
(A) Malignant mesenchymal neoplasia consisting of a vascular and solid pattern with an “epithelioid” aspect. (B) Bone tissue with marrow and malignant tumor with formation of vascular channels. (C) Bone spicules focally infiltrated by malignant vascular neoplasia.

The patient underwent a C6-C7 laminectomy and tumor resection with near total excision. Three specimens in formalin were sent to the pathology laboratory which showed malignant mesenchymal neoplasia consisting of a vascular and solid pattern with an “epithelioid” aspect highly pleomorphic, hyperchromatic, frequent mitosis, and multiple vascular channels, generating a multifocal cribriform appearance with extension to the bone and areas of necrosis (Figure 3A). Bone tissue with marrow and malignant tumor with formation of vascular channels and soft tissue were composed by skeletal muscle without alterations (Figure 3B). Bone spicules were focally infiltrated by malignant vascular neoplasia with dense connective tissue and adipose tissue without tumor (Figure 3C). Histopathological diagnosis of grade 2 epithelioid angiosarcoma with focal infiltration to C6-C7 lamina was made. The patient showed marked improvement 48 hours postoperatively with strength 5/5 of upper and lower limbs, patellar and Achilles reflex (2+), negative Babinski sign, preserved tactile and pain sensitivity.

## Discussion

The diagnosis of angiosarcoma is still a challenge, despite all of the technological advances in recent years, as they arise from the endothelium, the clinical presentation is ambiguous, and they can affect any part of the body [7]. The tumor usually presents in 60-70-year-old males, similar to our patient [8,9]. Spinal cord compression can arise from different identities, with spinal tumors being a known cause. Spinal tumors can be divided into extradural tumors (60% of the cases), intradural extramedullary tumors, and intradural intramedullary tumors [10]. Extradural tumors may grow into the epidural space and cause symptoms of spinal cord compression, as seen in our patient. To the best of our knowledge, no case has been reported so far in the literature of a deep soft tissue angiosarcoma causing spinal compression. Only reports of metastatic angiosarcoma to the cervical spine have been reported previously [11].

MRI is the gold standard for assessing spinal involvement which allows examining soft tissue, intervertebral disc, spinal cord, meninges, musculature, and ligaments. In this case, MRI was useful in determining the extent of the tumor before the surgery [12].

There are different treatment options for angiosarcoma; however, wide-margin surgical resection is the primary treatment of choice [1]. Due to the vascular nature of the tumor, patients are at a high risk of intraoperative bleeding, and our patient had approximately 1.5 L of blood loss. Because this tumor has a high rate of recurrence, radiotherapy is highly recommended [1]. A retrospective study by Scott et al. of 41 patients reported a five-year overall survival rate of 54% after radiotherapy. Tumor size of ≤5 cm and patients treated with a combination of wide surgical resection and radiotherapy were associated with a statistically significant predictor of survival (p = 0.0238) [13]. The patient in the presented case was referred for evaluation to determine if he was a candidate for radiosurgery.

## Conclusions

Angiosarcoma is a rare highly malignant tumor that can originate from any part of the body. As it remains a diagnostic challenge for the physician, a multidisciplinary approach is recommended. Owing to the aggressive nature of the lesion, early diagnosis is crucial. More studies are needed for early diagnosis, treatment, and prevention.
